# Association between platelet-to-albumin ratio and major depressive disorder: findings from a Chinese clinical cohort supported by population-based evidence

**DOI:** 10.3389/fpsyt.2026.1995336

**Published:** 2026-10-29

**Authors:** Kun Wang, Xiaofei Sun, Kun Li, Ying Fu, Yalan Zhang, Xun Zhang, Yong Yao

**Affiliations:** 1 Department of Psychiatry, Affiliated Hospital of West Anhui Health Vocational College, Lu’an, Anhui, China; 2 Department of Pathogenic Microbiology, School of Basic Medical Sciences, Anhui Medical University, Hefei, China; 3 Department of Medical Technology, West Anhui Health Vocational College, Lu’an, Anhui, China; 4 Clinical Laboratory, Affiliated Hospital of West Anhui Health Vocational College, Lu’an, Anhui, China

**Keywords:** inflammation, major depressive disorder, nutritional status, platelet indices, platelet-to-albumin ratio

## Abstract

**Background:**

Platelet dysfunction has been increasingly implicated in the pathophysiology of major depressive disorder (MDD), yet conventional platelet indices have shown inconsistent associations with the disease. The platelet-to-albumin ratio (PAR), a composite biomarker integrating inflammatory and nutritional status, has not been comprehensively investigated in relation to MDD across different populations. This study aimed to examine the association between PAR and depression using two independent cross-sectional cohorts.

**Methods:**

We analyzed data from a population-based NHANES cohort (n = 34,068) and a Chinese clinical cohort (n = 735). Depression in the NHANES cohort was assessed using the Patient Health Questionnaire-9 (PHQ-9), whereas MDD in the clinical cohort was diagnosed by psychiatrists according to DSM-5 criteria. Multivariable logistic and linear regression, subgroup analyses, and restricted cubic spline (RCS) models were used to examine the associations of platelet count (PLT), mean platelet volume (MPV), and PAR with depression. Bonferroni correction was applied to account for multiple comparisons in the regression analyses.

**Results:**

PAR was consistently and positively associated with depression across both cohorts after adjustment for potential confounders and Bonferroni correction. In the fully adjusted model, each unit increase in PAR was associated with higher odds of depression in both the NHANES cohort (OR = 1.047, 95% CI: 1.013–1.081, *P* = 0.006, Bonferroni-adjusted *P* = 0.018) and the clinical cohort (OR = 1.446, 95% CI: 1.267–1.659, *P* < 0.001, Bonferroni-adjusted *P* < 0.001). In contrast, PLT and MPV showed no consistent associations across the two cohorts. PAR was positively associated with depressive symptom severity in NHANES, whereas no significant association was observed in the clinical cohort. RCS analyses suggested an approximately linear association in NHANES but a nonlinear association in the clinical cohort. Stronger associations were observed among smokers, alcohol drinkers, and individuals without a partner in the clinical cohort.

**Conclusions:**

Across two independent cross-sectional cohorts with different population characteristics and outcome assessments, PAR showed a consistent positive association with depression, whereas conventional platelet indices did not. These findings suggest that PAR may provide additional information on depression-related inflammatory and nutritional alterations beyond individual platelet parameters. Prospective studies are warranted to clarify the temporal relationship and evaluate its potential clinical utility.

## 1 Introduction

Major depressive disorder (MDD), a psychiatric disorder characterized by persistent low mood, anhedonia, and suicidal ideation, is experiencing an increasing incidence worldwide (1). It is estimated that approximately 5% of adults and 20% of children and adolescents globally suffer from MDD, with this proportion steadily rising over time (2, 3). The COVID-19 pandemic has further exacerbated this trend, resulting in a 27.6% increase in MDD cases worldwide (4). MDD not only significantly reduces patients’ quality of life but also frequently co-occurs with various medical comorbidities, impaired social functioning, and a substantial family and societal burden (5, 6). Given the high prevalence of depression and the severe consequences of untreated cases, early diagnosis and effective intervention are crucial (7). However, the complex and multifactorial pathophysiology of MDD limits the clinical utility of individual biomarkers, which often fail to capture the heterogeneous biological alterations underlying the disorder (8, 9). therefore, identifying easily accessible composite biomarkers that better reflect the multifaceted nature of MDD has become an important research priority.

Platelets are increasingly recognized as a bridge linking psychiatric disorders, immune-related diseases, and coagulation dysfunction (10, 11). Although platelet-related biomarkers have attracted increasing attention in psychiatric research, findings regarding conventional platelet indices, including platelet count (PLT) and mean platelet volume (MPV), remain inconsistent across studies (12, 13). These inconsistencies suggest that single platelet parameters may not adequately capture the complex inflammatory and metabolic alterations associated with MDD. Consequently, composite biomarkers integrating multiple biological processes have emerged as a promising alternative. Among them, the platelet-to-albumin ratio (PAR), a novel composite biomarker, reflecting both inflammatory status and nutritional conditions, has demonstrated significant value in predicting prognosis across various inflammatory diseases (14–16).

Dysregulation of inflammatory processes plays a pivotal role in the pathophysiology of depression (17, 18), and platelets serve as key participants in both inflammation and immune responses (19, 20). Elevated platelet counts have been observed in various psychiatric disorders, including MDD (21). Conversely, albumin, an essential indicator of nutritional status, exhibits a significant association with MDD (22, 23). Studies have revealed that serum albumin levels are significantly lower in MDD patients compared to healthy controls and are inversely correlated with MDD severity (24, 25). Furthermore, serum albumin levels are closely associated with an increased risk of post-stroke depression in the elderly (26). By integrating platelet-related inflammatory activity and albumin-associated nutritional status, PAR may serve as a composite biomarker that captures depression-related biological alterations more comprehensively than conventional platelet indices alone.

In this study, we investigated the associations of PAR and other platelet-related parameters with depression across two complementary cross-sectional cohorts. We analyzed a large population-based survey and a Chinese clinical cohort with psychiatrist-confirmed DSM-5 diagnoses, and examined whether the associations were directionally consistent across these distinct population and clinical settings.

## 2 Methods

### 2.1 Study design and participants

This study employed a cross-sectional design using two complementary cohorts. The first was a large, population-based cohort derived from the National Health and Nutrition Examination Survey (NHANES), representing the general U.S. population. The second was a Chinese clinical cohort comprising participants with psychiatrist-confirmed MDD and healthy controls. The two cohorts differed in population characteristics and methods of depression assessment, allowing examination of the consistency of the observed associations across population-based and clinical settings.

For the population-based cohort, participants were selected from NHANES 2005-2020. Individuals aged <20 years, pregnant women, and participants with missing data on platelet-related parameters, albumin measurements, depressive assessment, or relevant covariates were excluded. After applying the predefined inclusion and exclusion criteria, a total of 34,068 participants were included in the final analysis. The detailed participant screening process is shown in **Figure 1**.

**Figure 1:**
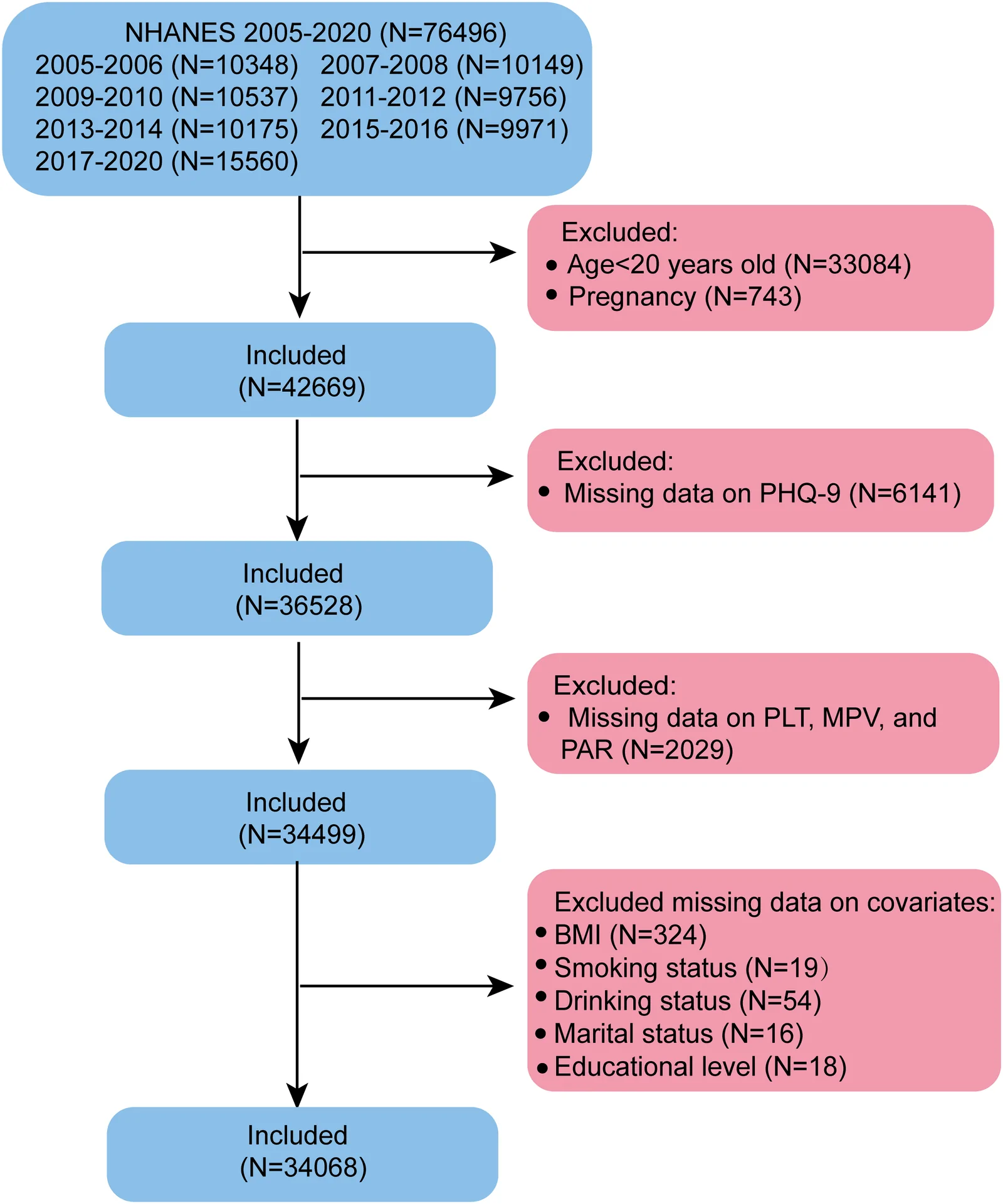
Flowchart of participant inclusion in the NHANES cohort.

For the Chinese clinical cohort, participants were recruited from the mental health center and the health examination center of the Affiliated Hospital of West Anhui Health Vocational College between 2019 and 2025. The clinical cohort included 735 participants, consisting of 308 patients with MDD and 427 healthy controls. The exclusion criteria included age <20 years, pregnancy, severe systemic diseases, acute infection, fever or allergic conditions within the previous two weeks, and incomplete clinical or laboratory information. Basic demographic characteristics, clinical information, and laboratory measurements were collected from all participants.

### 2.2 Exposure variables

In this study, PLT, MPV, and PAR were considered as exposure variables. In NHANES, blood samples were collected at mobile examination centers, and platelet-related parameters and serum albumin concentrations were measured according to standardized laboratory protocols. In the clinical cohort, participants were required to fast for at least 8 hours before venous blood samples were collected the following morning. The collected samples were immediately sent to the clinical laboratory for testing to ensure data accuracy and reliability. PAR was calculated by dividing platelet count by serum albumin concentration, serving as a composite indicator of platelet-related inflammatory activity and nutritional status.

### 2.3 Outcome variables

Depression status in the NHANES dataset was assessed using the Patient Health Questionnaire-9 (PHQ-9), a validated self-report screening instrument widely used for identifying depressive symptoms in population-based studies. A total PHQ-9 score ≥10 was used to define screening-positive depression, consistent with previous epidemiological studies (27, 28). In the Chinese clinical cohort, MDD was diagnosed by trained psychiatrists according to the Diagnostic and Statistical Manual of Mental Disorders, Fifth Edition (DSM-5) criteria. Depression severity was assessed using the 24-item Hamilton Depression Rating Scale (HAMD-24) (29). All patients with MDD had HAMD-24 scores ≥20, indicating clinically significant depressive symptoms (30, 31).

### 2.4 Covariates

Based on previous studies and available information from both cohorts, potential confounders including age, sex, education level, marital status, smoking status, alcohol consumption, and body mass index (BMI) were included in multivariable analyses. Age was categorized into two groups: 20–39 years and ≥40 years; education level was classified as college and non-college (32); and marital status was grouped as living with a partner or not (33). In NHANES, smoking status was defined as non-smoker (<100 cigarettes in a lifetime) or smoker (≥100 cigarettes) (34), and alcohol consumption was defined as non-drinker (<12 drinks in a lifetime) or drinker (≥12 drinks) (35). In the Chinese clinical cohort, smoking and alcohol consumption were categorized according to current smoking and drinking status. BMI was categorized into <25 kg/m², 25 to <30 kg/m², and ≥30 kg/m².

### 2.5 Statistical analysis

For baseline characteristic description, the normality of continuous variables was assessed using the Anderson–Darling test. Continuous variables were presented as the median (interquartile range) for non-normally distributed data, and categorical variables as the sample size (percentage). For NHANES, survey-weighted medians and interquartile ranges were calculated for continuous variables, and unweighted counts with survey-weighted percentages were reported for categorical variables. Between-group differences in continuous variables were assessed using survey-adjusted Wilcoxon rank-sum tests in the NHANES cohort and conventional Wilcoxon rank-sum tests in the Chinese clinical cohort. Categorical variables were compared using survey-adjusted chi-square tests in NHANES and Pearson’s chi-square tests in the clinical cohort.

To evaluate the associations between platelet-related parameters and depression-related outcomes, we constructed multivariable logistic regression models and multivariable linear regression models, respectively. Model 1 was an unadjusted crude model; Model 2 was adjusted for age, sex, education level, and marital status; and Model 3 was further adjusted for smoking status, alcohol consumption, and BMI. Bonferroni correction was applied separately within each cohort and regression model to account for multiple comparisons among PLT, MPV, and PAR. Both unadjusted and Bonferroni-adjusted *P*-values were reported for the regression analyses. In addition, subgroup analyses were conducted in both cohorts to examine potential differences across populations. Restricted cubic spline (RCS) regression was applied to further explore potential nonlinear relationships between platelet parameters and MDD.

Considering the complex sampling design of NHANES, MEC sample weights were recalculated according to the NHANES analytic guidelines, with each survey cycle weighted according to its respective survey duration. The adjusted combined-cycle weights, sampling strata, and primary sampling units were incorporated into the analyses to account for the complex survey design and obtain nationally representative estimates. All statistical analyses and visualizations were conducted using R software (v4.4.0). A two-sided *P*-value <0.05 was considered statistically significant.

## 3 Results

### 3.1 Baseline characteristics

This study included two independent cohorts. The NHANES cohort comprised 34,068 participants, including 31,074 without depression and 2,994 with screening-positive depression. The clinical cohort included 735 participants, consisting of 308 patients with MDD and 427 healthy controls. In NHANES, participants with depression were more likely to be female, have lower educational attainment, live without a partner, and have a history of smoking (all *P* < 0.001). In the clinical cohort, participants with MDD were more likely to have lower educational attainment (*P* = 0.048) and live without a partner (*P* < 0.001), whereas the sex distribution did not differ significantly between groups (*P* = 0.332).

Regarding laboratory parameters, albumin levels were significantly lower and PAR values were significantly higher in participants with depression in both cohorts (all *P* < 0.001). Platelet counts were significantly higher in the NHANES depression group (*P* < 0.001), but not in the clinical MDD group (*P* = 0.139). No significant differences in MPV were observed between depression and control groups in either cohort (NHANES: *P* = 0.443; clinical cohort: *P* = 0.502). Detailed baseline characteristics are presented in **Table 1**, and the distributions of PLT, MPV, and PAR in the clinical cohort are illustrated in **Figure 2**.

**Table 1:** Baseline characteristics of the study population.

Characteristic	NHANES			Clinical cohort		
	Non-MDD (n=31074)	MDD (n=2994)	*P*	Non-MDD (n=427)	MDD (n=308)	*P*
Age, years, n (%)			0.961			<0.001
20-39	10074 (35.31)	916 (35.38)		125 (29.27)	132 (42.86)	
≥40	21000 (64.69)	2078 (64.62)		302 (70.73)	176 (57.14)	
Sex, n (%)			<0.001			0.332
Female	15167 (49.57)	1884 (63.63)		289 (67.68)	219 (71.10)	
Male	15907 (50.43)	1110 (36.37)		138 (32.32)	89 (28.90)	
Educational level, n (%)			<0.001			0.048
Non-College	14068 (37.32)	1767 (51.48)		105 (24.59)	96 (31.17)	
College	17006 (62.68)	1227 (48.52)		322 (75.41)	212 (68.83)	
Living with a partner, n (%)			<0.001			<0.001
No	12088 (34.94)	1641 (51.68)		25 (5.85)	82 (26.62)	
Yes	18986 (65.06)	1353 (48.32)		402 (94.15)	226 (73.38)	
Smoking status, n (%)			<0.001			0.078
No	17488 (56.09)	1224 (38.55)		366 (85.71)	249 (80.84)	
Yes	13586 (43.91)	1770 (61.45)		61 (14.29)	59 (19.16)	
Drinking status, n (%)			0.004			0.487
No	4103 (10.14)	346 (8.45)		363 (85.01)	256 (83.12)	
Yes	26971 (89.86)	2648 (91.55)		64 (14.99)	52 (16.88)	
BMI (kg/m2), n (%)			<0.001			0.069
<25	8785 (29.36)	699 (26.37)		290 (67.92)	233 (75.65)	
25 to <30	10500 (33.66)	800 (26.02)		124 (29.04)	69 (22.40)	
≥30	11789 (36.98)	1495 (47.61)		13 (3.04)	6 (1.95)	
ALB (g/L), median (P25, P75)	43.00 (40.00,45.00)	42.00 (39.00,44.00)	<0.001	46.70 (44.90,48.40)	43.22 (41.50,45.30)	<0.001
PLT (×10^9^/L), median (P25, P75)	240.00 (205.00,283.00)	251.00 (212.00,296.00)	<0.001	228.00 (194.00,258.00)	233.00 (190.75,277.25)	0.139
MPV (fL), median (P25, P75)	8.10 (7.50,8.70)	8.10 (7.60,8.80)	0.443	9.99 (9.31,10.77)	9.90 (9.17,10.80)	0.502
PAR, median (P25, P75)	5.63 (4.74,6.72)	5.98 (5.02,7.28)	<0.001	4.89 (4.18,5.59)	5.36 (4.41,6.34)	<0.001

*P*-values were calculated using survey-adjusted chi-square tests or Wilcoxon rank-sum tests for NHANES, and Pearson's chi-square tests or Mann–Whitney U tests for the clinical cohort, as appropriate.

MDD, Major depressive disorder; BMI, body mass index; ALB, albumin; PLT, platelet count; MPV, mean platelet volume; PAR, platelet-to-albumin ratio.

**Figure 2:**
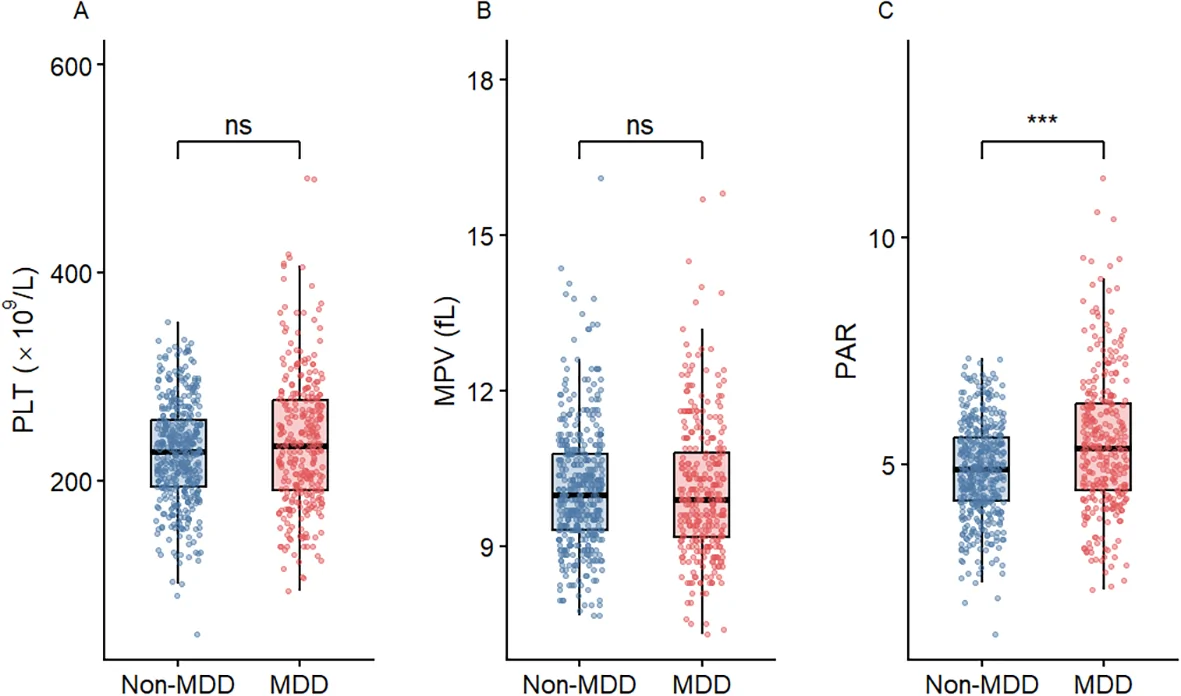
Boxplots of platelet-related biomarkers in Non-MDD and MDD participants from the clinical cohort. **(A)** PLT; **(B)** MPV; **(C)** PAR. Between-group differences were assessed using the Mann–Whitney U test. ns, not significant; ***P < 0.001. PLT, platelet count; MPV, mean platelet volume; PAR, platelet-to-albumin ratio.

### 3.2 Association between platelet-related parameters and MDD

Multivariable logistic regression analyses were performed to evaluate the associations of platelet-related parameters with depression across the two independent cohorts. In the NHANES cohort, PLT showed a modest association with depression in the crude and partially adjusted models; however, this association was attenuated after further adjustment. In contrast, PAR remained significantly associated with depression across all three models after Bonferroni correction. MPV was not significantly associated with depression.

A similar pattern was observed in the Chinese clinical cohort. PAR showed a consistent positive association with clinically diagnosed MDD across all adjustment models after Bonferroni correction, whereas PLT was significant only in the unadjusted model and MPV showed no significant association. In the fully adjusted Model 3, PAR was significantly associated with depression in both cohorts, with an OR of 1.047 (95% CI: 1.013–1.081, *P* = 0.006, Bonferroni-adjusted *P* = 0.018) in the NHANES cohort and 1.446 (95% CI: 1.267–1.659, *P* < 0.001, Bonferroni-adjusted *P* < 0.001) in the Chinese clinical cohort (**Table 2**).

**Table 2:** Association between platelet-related parameters and MDD.

Model	NHANES			Clinical cohort		
	OR (95%CI)	*P*	*P* (Bonferroni-adjusted)	OR (95%CI)	*P*	*P* (Bonferroni-adjusted)
Model 1						
PLT	1.002 (1.002,1.003)	<0.001	<0.001	1.003 (1.001,1.006)	0.015	0.045
MPV	1.033 (0.962,1.111)	0.367	1.000	0.986 (0.877,1.109)	0.819	1.000
PAR	1.135 (1.103,1.169)	<0.001	<0.001	1.455 (1.291,1.649)	<0.001	<0.001
Model 2						
PLT	1.001 (1.000,1.002)	0.002	0.006	1.002 (0.999,1.005)	0.269	0.807
MPV	1.018 (0.948,1.092)	0.625	1.000	1.021 (0.903,1.155)	0.735	1.000
PAR	1.080 (1.047,1.114)	<0.001	<0.001	1.408 (1.237,1.610)	<0.001	<0.001
Model 3						
PLT	1.001 (1.000,1.002)	0.093	0.279	1.002 (0.999,1.005)	0.167	0.501
MPV	1.007 (0.937,1.082))	0.854	1.000	1.005 (0.887,1.137)	0.943	1.000
PAR	1.047 (1.013,1.081)	0.006	0.018	1.446 (1.267,1.659)	<0.001	<0.001

Model 1 included no covariate adjustments. Model 2 was adjusted for age, sex, educational level, and marital status. Model 3 was further adjusted for smoking status, drinking status, and BMI. Bonferroni correction was applied separately within each cohort and model to account for multiple comparisons among PLT, MPV, and PAR (three comparisons per model). Both unadjusted and Bonferroni-adjusted *P*-values are presented.

PLT, platelet count; MPV, mean platelet volume; PAR, platelet-to-albumin ratio.

### 3.3 Subgroup analysis of the association between PAR and MDD

Subgroup analyses further revealed differences in the association between PAR and MDD across subgroups in the two cohorts (**Figure 3**). In the NHANES cohort, no significant differences were observed in the PAR–MDD association across subgroups. In contrast, in the clinical cohort, the association between PAR and MDD appeared more pronounced among smokers, drinkers, and individuals without a partner.

**Figure 3:**
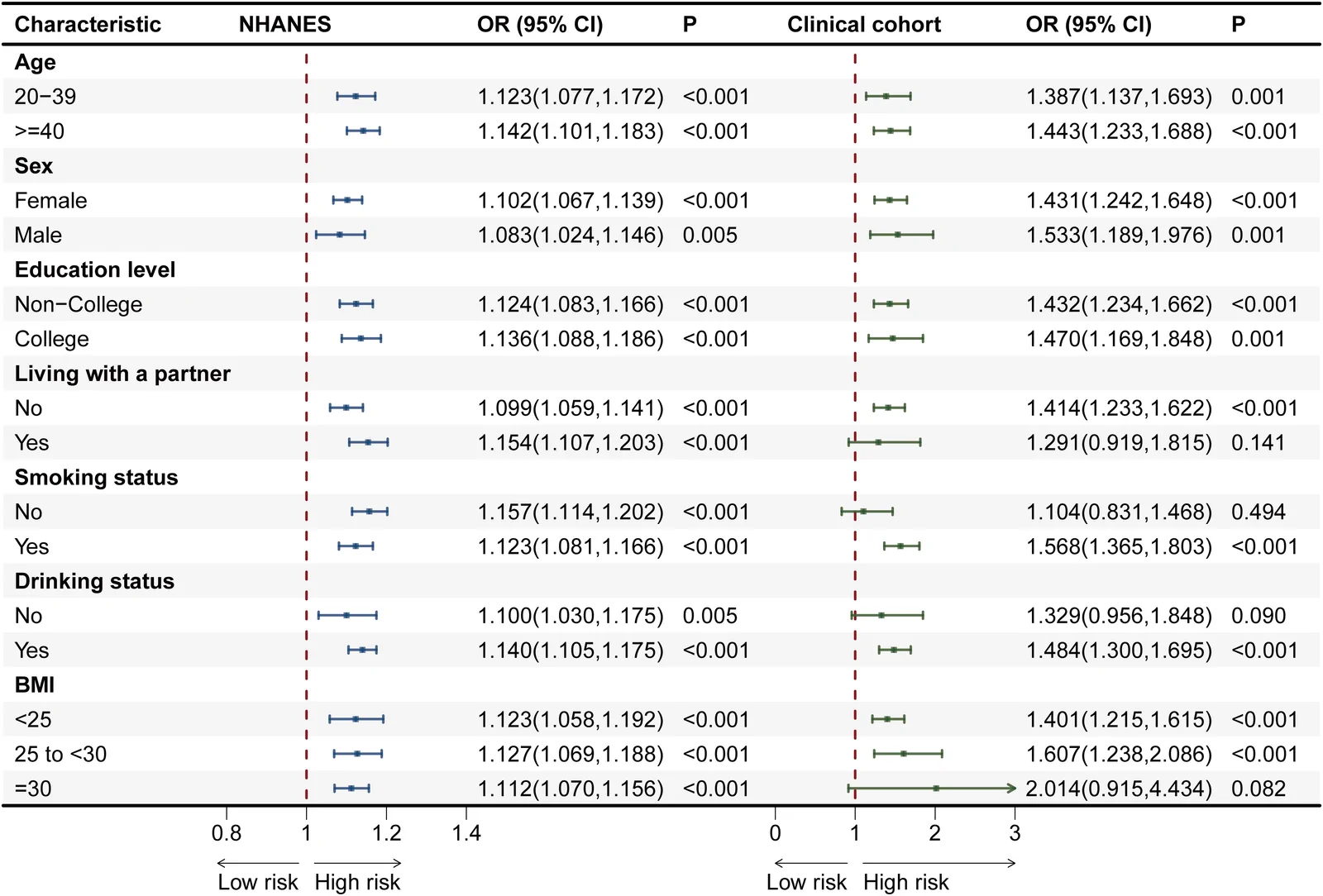
Subgroup analysis of the association between PAR and MDD. Subgroup analyses were exploratory, and *P*-values were not adjusted for multiple comparisons.

### 3.4 Association between platelet-related parameters and MDD severity

Multivariable linear regression analyses were further performed to investigate the associations between platelet-related parameters and depressive symptom severity. In the NHANES cohort, PAR remained positively associated with PHQ-9 scores in the fully adjusted Model 3 (β = 0.070, 95% CI: 0.031–0.109, *P* < 0.001, Bonferroni-adjusted *P* = 0.001). Although PLT was significantly associated with PHQ-9 scores before correction (*P* = 0.047), this association was no longer significant after Bonferroni correction (adjusted *P* = 0.141). MPV was not significantly associated with PHQ-9 scores. In contrast, in the Chinese clinical cohort, PAR showed a negative but statistically nonsignificant regression coefficient for HAMD-24 scores in Model 3 (β = −0.335, 95% CI: −0.793 to 0.124, *P* = 0.152, Bonferroni-adjusted *P* = 0.456). Neither PLT nor MPV was significantly associated with HAMD-24 scores in the clinical cohort (**Table 3**).

**Table 3:** Association between platelet parameters and MDD severity.

Model	NHANES			Clinical cohort		
	β (95%CI)	*P*	*P* (Bonferroni-adjusted)	β (95%CI)	*P*	*P* (Bonferroni-adjusted)
Model 1						
PLT	0.004 (0.003,0.005)	<0.001	<0.001	-0.008 (-0.019,0.002)	0.117	0.351
MPV	0.047 (-0.021,0.114)	0.174	0.552	0.463 (-0.049,0.974)	0.076	0.228
PAR	0.220 (0.183,0.258)	<0.001	<0.001	-0.383 (-0.824,0.059)	0.089	0.267
Model 2						
PLT	0.002 (0.001,0.003)	<0.001	<0.001	-0.009 (-0.020,0.002)	0.106	0.318
MPV	0.022 (-0.044,0.089)	0.508	1.000	0.448 (-0.064,0.961)	0.086	0.258
PAR	0.129 (0.090,0.168)	<0.001	<0.001	-0.399 (-0.849,0.051)	0.082	0.246
Model 3						
PLT	0.001 (0.000,0.002)	0.047	0.141	-0.007 (-0.018,0.003)	0.179	0.537
MPV	0.000 (-0.066,0.066))	0.998	1.000	0.380 (-0.142,0.902)	0.153	0.459
PAR	0.070 (0.031,0.109)	<0.001	0.001	-0.335 (-0.793,0.124)	0.152	0.456

Model 1 included no covariate adjustments. Model 2 was adjusted for age, sex, educational level, and marital status. Model 3 was further adjusted for smoking status, drinking status, and BMI. Bonferroni correction was applied separately within each cohort and model to account for multiple comparisons among PLT, MPV, and PAR (three comparisons per model). Both unadjusted and Bonferroni-adjusted *P*-values are presented.

PLT, platelet count; MPV, mean platelet volume; PAR, platelet-to-albumin ratio.

### 3.5 Analysis of RCS regression

RCS analyses were performed to further characterize the potential dose–response relationships between platelet-related parameters and depression. After adjustment for covariates, PLT and PAR showed significant overall associations with depression in the NHANES cohort, while the tests for nonlinearity were not statistically significant, suggesting approximately linear relationships. In the Chinese clinical cohort, both PLT and PAR demonstrated significant nonlinear associations with MDD, with inflection points at 226 and 4.57, respectively. No significant association was observed between MPV and depression in either cohort (**Figure 4**).

**Figure 4:**
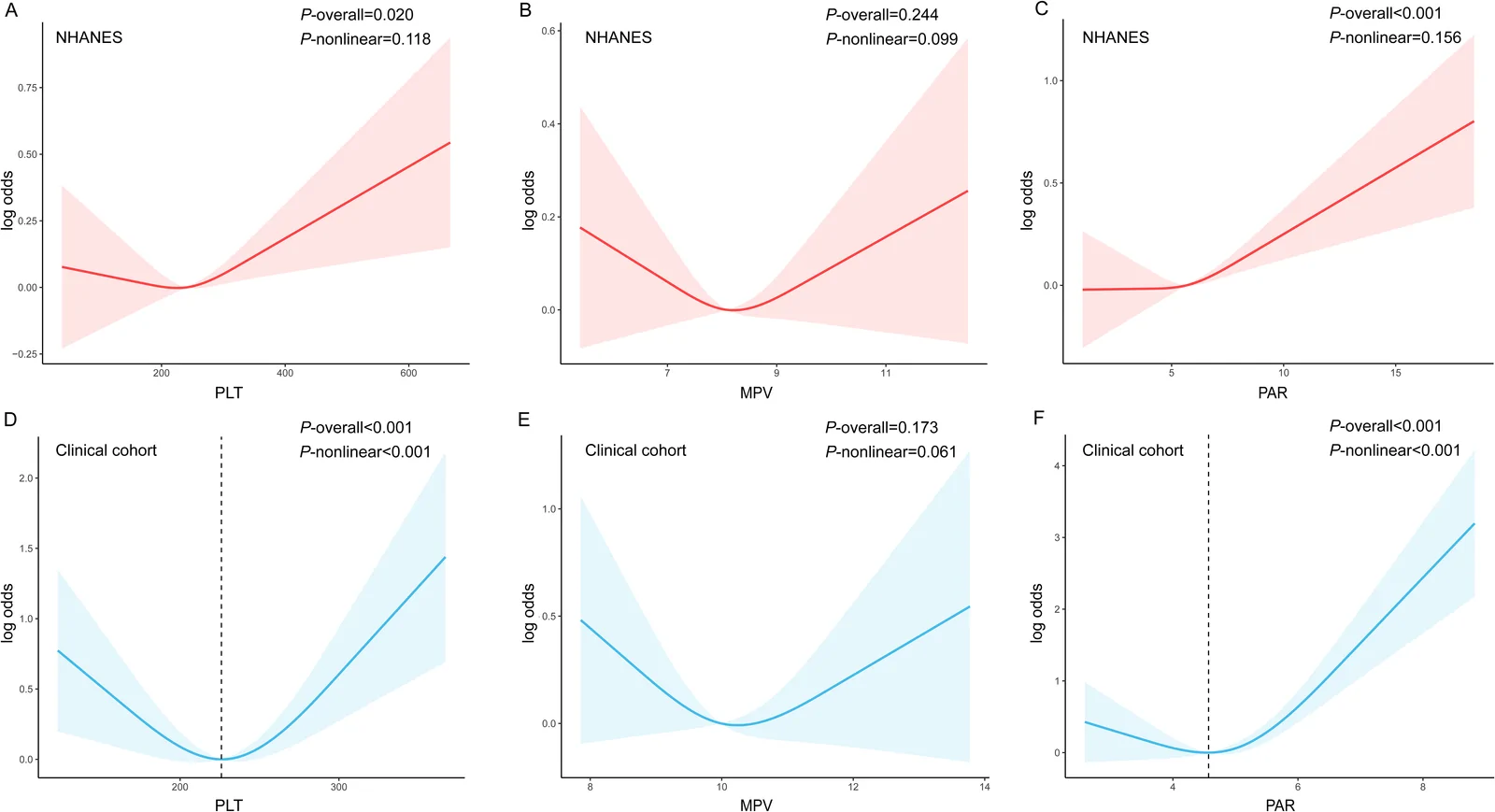
Restricted cubic spline plots adjusted for covariates. **(A)** PLT and MDD in NHANES; **(B)** MPV and MDD in NHANES; **(C)** PAR and MDD in NHANES; **(D)** PLT and MDD in clinical cohort; **(E)** MPV and MDD in clinical cohort; **(F)** PAR and MDD in clinical cohort. Models were adjusted for age, sex, educational level, marital status, smoking status, drinking status, and BMI. PLT, platelet count; MPV, mean platelet volume; PAR, platelet-to-albumin ratio.

## 4 Discussion

In this study, we investigated the associations between platelet-related parameters and depression using two independent cross-sectional cohorts with different population characteristics and assessment approaches. Across both cohorts, PAR showed a consistent positive association with depression after adjustment for potential confounders and Bonferroni correction, whereas conventional platelet indices, including PLT and MPV, did not demonstrate consistent associations. Furthermore, PAR was positively associated with depressive symptom severity in the population-based cohort, but no significant association was observed in the clinical cohort. The dose–response relationships between PAR and depression differed between cohorts, with an approximately linear pattern observed in the population-based cohort and a nonlinear pattern observed in the clinical cohort. These findings suggest that PAR may better capture depression-related biological alterations than individual platelet parameters, although further longitudinal studies are required to determine its clinical significance.

MDD is a complex psychiatric disorder involving multiple biological pathways, among which neuroinflammation and immune dysregulation have received increasing attention (36, 37). The neuroinflammatory hypothesis suggests that persistent inflammatory activation may contribute to depressive symptoms through altered neurotransmitter metabolism, impaired neuroplasticity, and hypothalamic–pituitary–adrenal (HPA) axis dysfunction (38, 39). Platelets, traditionally recognized for their role in hemostasis, are increasingly considered important mediators of immune and inflammatory responses. Activated platelets can release various inflammatory mediators and participate in systemic immune regulation (40, 41). Therefore, platelet-related alterations may reflect part of the inflammatory abnormalities associated with depression.

However, individual platelet parameters may not sufficiently represent the complex biological changes underlying depression. Previous studies have reported inconsistent associations between PLT, MPV, and depressive disorders (13, 42). In contrast, PAR integrates information from platelet abundance and serum albumin concentration, thereby simultaneously reflecting platelet-related inflammatory activity and nutritional-inflammatory status. Albumin, as a negative acute-phase protein, decreases during systemic inflammatory activation and has been associated with adverse clinical outcomes in several inflammatory and psychiatric conditions (43, 44). Reduced albumin levels may indicate impaired antioxidant capacity and increased susceptibility to inflammatory stress (45, 46). Therefore, the combined assessment of platelet and albumin levels through PAR may provide a more comprehensive representation of inflammation-related biological alterations than either parameter alone.

Although previous studies have separately investigated platelet parameters and albumin levels in relation to depression, the association between PAR and depression has remained insufficiently explored (47, 48). I In the present study, PAR showed a more consistent association with depression across two independent cohorts compared with PLT and MPV. Interestingly, the dose–response relationship between PAR and depression differed between the population-based and clinical cohorts. The nonlinear pattern observed in the clinical cohort may reflect the complex biological characteristics of patients with clinically diagnosed MDD, who may differ in disease severity, treatment histories, and inflammatory or metabolic states. In contrast, the population-based cohort included individuals with a broader range of depressive symptoms, which may contribute to the relatively linear association observed. Differences in sample size between the NHANES cohort (n = 34,068) and the clinical cohort (n = 735) may also have influenced the precision of effect estimates and the statistical power to detect associations. These differences highlight the importance of considering population characteristics and clinical context when interpreting associations between biological markers and depression.

Notably, the association between PAR and depressive symptom severity was not consistent across the two cohorts. In NHANES, PAR was positively associated with PHQ-9 scores after full adjustment (β = 0.070, 95% CI: 0.031–0.109, Bonferroni-adjusted *P* = 0.001). However, in the Chinese clinical cohort, the regression coefficient for PAR and HAMD-24 scores was negative but not statistically significant (β = −0.335, 95% CI: −0.793 to 0.124, Bonferroni-adjusted *P* = 0.456). This difference may partly reflect the distinct depression assessment instruments and study populations. PHQ-9 is a self-reported screening instrument assessing depressive symptoms in the general population, whereas HAMD-24 is a clinician-rated scale used to evaluate symptom severity in patients with clinically diagnosed MDD. Moreover, all patients with MDD in the clinical cohort had HAMD-24 scores ≥20, potentially restricting the range of symptom severity compared with the population-based cohort. The smaller clinical sample size and potential differences in treatment exposure, disease duration, and underlying inflammatory or metabolic conditions may have further contributed to the inconsistent findings. Importantly, the nonsignificant negative coefficient in the clinical cohort should not be interpreted as evidence of an inverse association. These findings suggest that although PAR was consistently associated with depression status, its relationship with symptom severity may vary across clinical settings and requires further investigation.

The mechanisms underlying the association between PAR and depression remain unclear. Several potential pathways may explain this relationship. First, chronic inflammatory activation or nutritional impairment may lead to increased platelet activation and reduced albumin levels, resulting in elevated PAR and potentially contributing to depression through immune-mediated mechanisms (49, 50). Second, depression itself may influence inflammatory and metabolic states through chronic psychological stress, altered hypothalamic–pituitary–adrenal axis activity, changes in dietary patterns, reduced physical activity, and other behavioral factors, which may subsequently affect platelet and albumin levels (13, 51). Therefore, the observed association between PAR and depression may represent a bidirectional relationship rather than a simple causal pathway. Longitudinal studies are needed to clarify the temporal sequence and underlying mechanisms.

Subgroup analyses suggested that the association between PAR and depression appeared more pronounced among smokers, alcohol drinkers, and individuals without a partner in the clinical cohort. These findings may indicate that lifestyle-related inflammatory burden and psychosocial factors could influence the relationship between PAR and depression (52, 53). However, these subgroup findings should be interpreted cautiously because they were exploratory and require confirmation in larger populations.

This study has several limitations. First, the cross-sectional design prevents determination of the temporal relationship between elevated PAR and depression, and causal conclusions cannot be drawn. Second, the two cohorts differed substantially in population characteristics, including geographic background, population source, and depression assessment methods. The NHANES cohort represented a general population-based sample assessed using PHQ-9 screening, whereas the clinical cohort consisted of patients with psychiatrist-confirmed MDD. These differences, together with the substantially smaller clinical sample size, may contribute to the variation in effect estimates and limit direct comparability between cohorts, particularly for analyses of depressive symptom severity. Third, the clinical cohort was derived from a single medical center, and selection bias cannot be completely excluded. In addition, information regarding some potential confounders, including inflammatory biomarkers such as CRP and IL-6, medication exposure, nutritional status, and chronic disease burden, was not consistently available and may have resulted in residual confounding. Differences in the definitions of smoking and alcohol consumption between cohorts may also limit direct comparability. Fourth, subgroup analyses were exploratory, and further studies are needed to verify whether specific populations show stronger associations. Finally, the generalizability of the findings requires confirmation in larger, multicenter, and longitudinal cohorts.

## 5 Conclusion

This study investigated the associations between platelet-related parameters and depression across two complementary cross-sectional cohorts with different population characteristics and assessment approaches. PAR showed a consistent positive association with depression in both the population-based cohort and the Chinese clinical cohort, whereas conventional platelet indices, including PLT and MPV, demonstrated less consistent associations. However, the association between PAR and depressive symptom severity was significant only in the population-based cohort. These findings suggest that PAR, as a composite biomarker integrating platelet-related inflammatory activity and nutritional status, may provide additional biological information beyond individual platelet parameters. Nevertheless, given the cross-sectional design, the temporal relationship and causal mechanisms between PAR and depression remain unclear. Further longitudinal studies and clinical investigations are warranted to clarify these associations and evaluate the potential clinical utility of PAR as an adjunctive biomarker for depression.

## Data Availability

The raw data supporting the conclusions of this article will be made available by the authors, without undue reservation.
